# Diversification of the *vacAs1m1* and *vacAs2m2* Strains of *Helicobacter pylori* in *Meriones unguiculatus*

**DOI:** 10.3389/fmicb.2016.01758

**Published:** 2016-11-08

**Authors:** Sandra Mendoza-Elizalde, Nancy K. Arteaga-Resendiz, Pedro Valencia-Mayoral, Raúl C. Luna, Sarbelio Moreno-Espinosa, Francisco Arenas-Huertero, Gerardo Zúñiga, Norma Velázquez-Guadarrama

**Affiliations:** ^1^Laboratorio de Infectología, Departamento de Infectologia, Hospital Infantil de México Federico GómezCiudad de México, Mexico; ^2^Posgrado en Ciencias Químicobiológicas, Escuela Nacional de Ciencias Biológicas, Instituto Politécnico NacionalCiudad de México, Mexico; ^3^Laboratorio de Variación Biológica y Evolución, Departamento de Zoología, Escuela Nacional de Ciencias Biológicas, Instituto Politécnico NacionalCiudad de México, Mexico; ^4^Posgrado en Ciencias en Biomedicina y Biotecnología Molecular, Escuela Nacional de Ciencias Biológicas, Instituto Politécnico NacionalCiudad de México, Mexico; ^5^Dirección de Planeación, Hospital Infantil de México Federico GómezCiudad de México, Mexico; ^6^Bioterio, Hospital Infantil de México Federico GómezCiudad de México, Mexico; ^7^Laboratorio de Investigación en Patología Experimental, Hospital Infantil de México Federico GómezCiudad de México, Mexico

**Keywords:** *H. pylori*, *Meriones unguiculatus*, animal model, diversification of genotypes, natural chimera, eBURST, PHYLOViZ

## Abstract

The bacterium *Helicobacter pylori* exhibits great genetic diversity, and the pathogenic roles of its virulence factors have been widely studied. However, the evolutionary dynamics of *H. pylori* strains during stomach colonization are not well-characterized. Here, we analyzed the microevolutionary dynamics of the toxigenic strain *vacAs1m1*, the non-toxigenic strain *vacAs2m2*, and a combination of both strains in an animal model over time. *Meriones unguiculatus* were inoculated with the following bacteria: group 1-toxigenic strain *vacAs1m1/cagA+/cagE+/babA2+*; ST181, group 2-non-toxigenic strain *vacAs2m2/cagA+/cagE+/babA2+*; ST2901, and group 3-both strains. The gerbils were euthanized at different time points (3, 6, 12, and 18 months). In group 1, genetic alterations were observed at 6 and 12 months. With the combination of both strains, group 3 also exhibited genetic alterations at 3 and 18 months; moreover, a chimera, *vacA m1-m2*, was detected. Additionally, four new sequence types (STs) were reported in the PubMLST database for *H. pylori*. Synonymous and non-synonymous mutations were analyzed and associated with alterations in amino acids. Microevolutionary analysis of the STs (PHYLOViZ) identified in each group revealed many mutational changes in the toxigenic (*vacAs1m1*) and non-toxigenic (*vacAs2m2*) strains. Phylogenetic assessments (eBURST) did not reveal clonal complexes. Our findings indicate that the toxigenic strain, *vacAs1m1*, and a combination of toxigenic and non-toxigenic strains acquired genetic material by recombination. The allelic combination, *vacAs2m1*, displayed the best adaptation in the animal model over time, and a chimera, *m1-m2*, was also identified, which confirmed previous reports.

## Introduction

*Helicobacter pylori*, a well-known member of the human microbiota, has a global distribution that is related to the migration of *Homo sapiens* over the past 60,000 years (Morelli et al., 2010; Moodley et al., 2012). *H. pylori* is a Gram-negative spiral bacterium that is associated with the development of peptic ulcers as well as some types of gastric lymphomas and gastric adenocarcinomas in humans (Mbulaiteye et al., 2009; Testerman and Morris, 2014).

The evolution of distinct genetic prototypes in *H. pylori* is linked to different human ethnic groups worldwide, supporting the presence of genetic mechanisms that have permitted rapid adaptation in human populations (Linz et al., 2007). High mutation rates and frequent inter-strain exchanges of genetic material that occur during infection are responsible for the extreme variation and genetic diversity among *H. pylori* strains (Blaser and Atherton, 2004; Suerbaum and Josenhans, 2007). Additionally, transmission mainly occurs through direct human-to-human contact, and single or multiple strains of *H. pylori* can colonize and recolonize a host to increase its variability (Brown, 2000; Frenck and Clemens, 2003). Consequently, mutations, inter-strain genetic exchange and the mode of transmission appear to account for the capacity of *H. pylori* to colonize different habitats in the stomach, and its indirect and direct interactions with the human host trigger different selective pressures that regulate the presence of strains in this changing habitat (Raymond et al., 2004; Kivi et al., 2007; Costa et al., 2009; Secka et al., 2011). Researchers have hypothesized that selective pressures that determine the presence of *H. pylori* in the stomach operate on three types of bacterial genes (e.g., genes that affect intrabacterial mutations, DNA uptake, repair and recombination; genes that favor bacteria–bacteria interactions; and genes that influence bacterial properties, such as adherence and immune responses that modulate interactions with the host) (Gangwer et al., 2010; Tanih et al., 2011).

The dynamics of *H. pylori* genotypes during stomach colonization are unknown because the successful establishment of these strains is an inadvertent process. A model that potentially explains the genotypic evolution of *H. pylori* in its human host assumes that strains with genotype *vacA*+/*cagA*+/*babA*+ are at a higher “fitness peak” (Montecucco and Rappuoli, 2001). These virulence genes encode proteins (i.e., VacA, CagA, and BabA) that help the bacteria to adhere and persist in the gastric epithelium by modifying and altering apical and cell junctions (Wroblewski et al., 2010); i.e., the *vacAs1m1* allelic combination is capable of producing the VacA toxin (which induces vacuolation of gastric epithelial cells), whereas the *vacAs2m2* allelic combination produces low amounts or none of the VacA toxin (Atherton et al., 1995; Letley and Atherton, 2000). Consequently, inactivation of any of these factors can shift the fitness of the strains. *H. pylori* can live in the stomach of an individual for many years, so it is possible that strains may emerge with *vacA+/-. cag+/-*, or *babA+/-* genotypes, and strains with the genotype *cag*- or *cag+* can be isolated from the same patient. However, these strains likely cannot survive long because of the high recombination rates observed for this bacterium. Thus, only those bacteria that are efficient over long durations and engage in person-to-person transmission are thought to govern the evolution of *H. pylori* (Montecucco and Rappuoli, 2001; Prouzet-Mauleon et al., 2005).

Genetic alterations that are produced during the microevolution of *H. pylori* have not been studied because they can only be detected during the transition phase, i.e., after the passage of an *in vitro* strain (culture) to an *in vivo* (animal) setting or during colonization of a host that is not infected (Ferrero and Jenks, 2001). Animals that are infected (e.g., Rhesus monkeys, mice, and gerbils) with strains of known genotypes provide experimental models (Peek, 2008; Behrens et al., 2013; Linz et al., 2014) that can be used to follow the evolution of these strains *in vivo*, from the initial inoculation until the definitive establishment of the strain (Morelli et al., 2010; Linz et al., 2014). In the present study, we analyzed the evolutionary dynamics of the toxigenic strain, *vacAs1m1*, and the non-toxigenic strain, *vacAs2m2*, separately and together in an animal model over time.

## Materials and Methods

### Animal Model

The 8-week-old Mongolian gerbils (*Meriones unguiculatus* Hsd:MON, Harlan Teklad, Madison, WI, USA) used in this study were housed under specific pathogen-free conditions in plastic metabolic cages to prevent coprophagy under standard laboratory conditions (i.e., room temperature, 23 ± 2°C; relative humidity 40–60%; and a 12-h light–dark cycle). Free access to a standard diet (special rodent food; Harlan Teklad, Madison, WI, USA) and sterilized tap water were provided. The Ethics, Biosafety and Scientific committees at the Health Institute approved the experiment.

The three groups of gerbils included five animals each one, ensuring that three animal would present *H. pylori* infection (Velazquez-Guadarrama et al., 2007). Gerbils were inoculated intragastrically with 500 mL NaHCO_3_ (0.2 M), and 1 h later with a bacterial suspension of different genotypes of *H. pylori* [6 × 10^8^ colony forming units (CFUs)/mL]. For 1 week, group 1 was inoculated with the toxigenic strain, *vacAs1m1/cagA+/cagE+/babA2+*; group 2 received the non-toxigenic strain, *vacAs2m2/cagA+/cagE+/babA2+*; and group 3 received both the toxigenic strain, *vacAs1m1*, and the non-toxigenic strain, *vacAs2m2*. The gerbils were fasted 18 h prior to the first inoculation until the end of the fifth inoculation. The *H. pylori* strains used in this study included reference strain 26695 (positive control for the *vacAs1m1* genotype) and clinical strain 174F2 (positive control for the *vacAs2m2* genotype). Control animals received saline alone. The gerbils were euthanized at 3, 6, 12, and 18 months by cervical dislocation under anesthesia to harvest the stomach. Assuming that the *H. pylori* generation time is at least 3 h (Jiang and Doyle, 2000; Joo et al., 2010), the generation numbers (G) achieved at these months were 654 G, 1309 G, 2617 G, and 3926 G, respectively. The stomach was dissected along the greater curvature and washed with phosphate-buffered saline (PBS pH 7.4, 0.01 M). It was then divided longitudinally into parts and macerated with Brucella broth (BD BBL) in a final volume of 200 μL. Next, 10 μL was used for a urease test, and 10 μL (STOCK) was inoculated in Casman agar plates (BD BBL, Sparks, MD, USA) with or without antibiotics (3 mg/mL vancomycin, 5 mg/mL trimeptoprim, and 2 mg/mL amphotericin B). Additionally, 170 μL was used for serial dilutions (1:10, 1:100, 1:1,000, and 1:10,000) on Casman agar plates with and without antibiotics. The plates were grown under microaerophilic conditions (5% O_2_, 5% CO_2_, 85% N_2_, and 10% humidity) at 37°C for 7–14 days. The control group was compared with the infected groups.

### Isolation and Identification of *H. pylori*

Bacterial isolation was performed using 10 presumptive colonies of *H. pylori* for each dilution of the different generations. Bacterial identification was based on colony morphology, Gram staining, and tests for urease, catalase, and oxidase. *H. pylori* was stored at -70°C in 1.5 mL Brucella broth (BD BBL) supplemented with 10% fetal bovine serum and 25% glycerol.

### Detection of Virulence Genes by PCR

Genomic DNA was extracted from a section of the stomach of gerbils, and colonies of *H. pylori* were isolated using a Wizard Genomic DNA Purification kit (Promega, Madison, WI, USA) according to the manufacturer’s instructions with slight modifications of the incubation times. The DNA was quantified in an Epoch Microplate Spectrophotometer (BioTek, software Gen5^TM^, Winooski, VT, USA), and the DNA integrity was evaluated by electrophoresis using 1% agarose gels. *H. pylori* was identified in gastric tissue and isolates based on the presence of the *glmM* gene (Smith et al., 2004). The *vacA* (*s1, s2, m1*, and *m2*), *cagA, cagE*, and *babA2* genes were amplified by PCR using the conditions described by Atherton et al. (1995), Mizushima et al. (2001), and Kauser et al. (2004). Amplification was performed in a reaction volume of 25 μL Master Mix (Promega) containing 100 ng bacterial DNA, 2.5 mM MgCl_2_, 10 mM dNTPs, 2 U Taq DNA polymerase, 20 pmol each primer and nuclease-free water in a Thermo Hybrid thermal cycler (PCR Express, Emeryville, CA, USA). The PCR products were separated by electrophoresis using 1% agarose gels at 80 V, followed by staining with ethidium bromide and imaging under UV illumination (ChemiDoc Transilluminator, Bio-Rad, Hercules, CA, USA). DNA from reference strain 26695 was included as a positive control.

### Multilocus Sequence Typing (MLST)

Internal fragments were amplified and sequenced in both directions for seven housekeeping genes [*mutY*, HP0142, specific adenine glycosylase A/G; *ureI*, HP0071, urea transporter; *atpA*, HP1134, ATP synthase F1 α subunit; *efp*, HP0177, elongation factor P (EF-P); *ppa*, HP0620, inorganic pyrophosphatase; *trpC*, HP1279, indole-3-glycerol phosphate synthase; and *yphC*, HP0834, GTPase], as reported in previous studies of *H. pylori* (Lundin et al., 2005; Kivi et al., 2007). The PCR conditions were as follows: 35 cycles of 94°C for 15 s, 55–62°C for 30 s, and 72°C for 1.5 min and a final extension at 72°C for 5 min (Achtman et al., 1999). The PCR products were purified using ExoSAP-IT^®^ (Affymetrix, Cleveland, OH, USA) according to the manufacturer’s recommendations. The purified products were sequenced using a BigDye Terminator v3.1 Cycle Sequencing kit with an ABI 3130 Genetic Analyzer (Applied Biosystems, Foster City, CA, USA).

### Bioinformatics and Phylogenetic Analyses

The sequences of the seven loci were aligned using ClustalX v2 (Larkin et al., 2007), edited with Seaview v4.2.5 (Gouy et al., 2010) and FinchTV V.1.4.0 Software (Geospiza, Inc.), and compared with those of all known alleles from *H. pylori* deposited in the PubMLST database^1^. Each strain was defined based on the presence of alleles of the seven genes (allelic profile); every allelic profile was defined as a sequence type (ST) (Feil et al., 2004; Vázquez and Berrón, 2004). To establish the open reading frame of each protein, the nucleotide sequences of the different STs from each housekeeping gene were translated into amino acid sequences using the translate tool in ExPASy^2^. To determine the presence of synonymous and non-synonymous mutations in different positions of the seven housekeeping genes, we used DnaSP v5.10^3^ (Librado and Rozas, 2009).

The clonal relationship among strains of *H. pylori* was determined using the PHYLOViZ platform^4^. PHYLOViZ infers evolutionary descent patterns among allelic profiles using the goeBURST algorithm and a full Minimal Spanning Tree (MST)-like approach (Francisco et al., 2009). The phylogenetic relationships among the strains was determined using the eBURST algorithm^5^, which subdivided large multilocus sequence typing (MLST) datasets into non-overlapping groups of related STs or clonal complexes to discern the location of the most parsimonious isolates within groups or clonal complexes from the predicted founder (Feil et al., 2004). In addition, the eBURST algorithm explores the diversification of strains and can provide evidence for the emergence of clones of particular clinical relevance.

The nucleotide sequences (alleles) and STs found in this study were deposited in the PubMLST database for *H. pylori*^1^ (Jolley et al., 2004). The accession numbers for each gene were as follows: *atpA* **2358**, **2470**; *efp* **2228**, **2354**; *mutY* **2391**; *ppa* **2252**; *trpC* **2503**, **2512**; *ureI* **2474**; and *yhpC* **2583**, and for STs: **ST2901**, **ST2902**, **ST2903**, **ST2904**, and **ST2905**.

## Results

**FIGURE 1:**
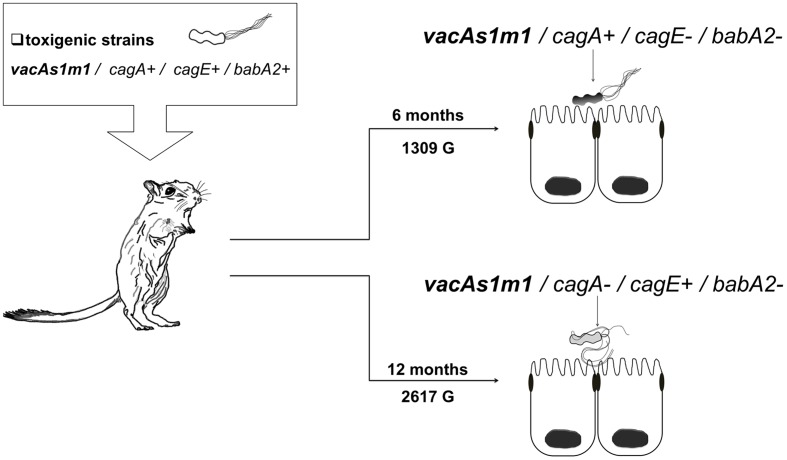
***Helicobacter pylori* genotypes identified with the group 1 toxigenic strain (*vacAs1m1*) in the animal model, *Meriones unguiculatus*.** Alterations in the *cag*-PAI and *babA2* genes occurred at 6 (1309 G) and 12 (2617 G) months.

**FIGURE 2:**
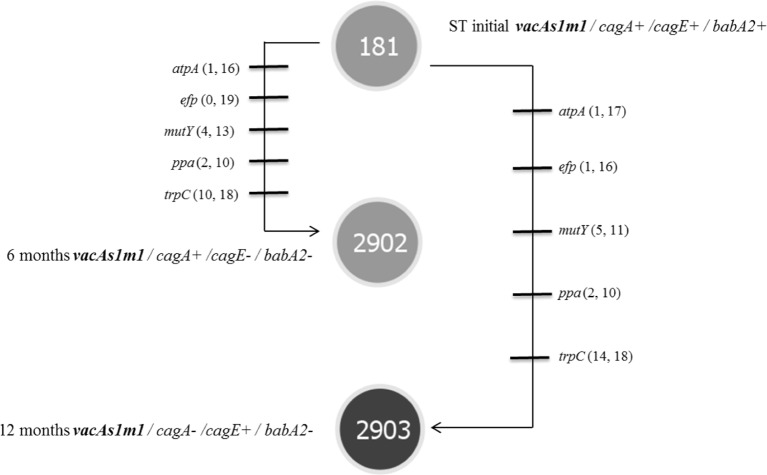
**Microevolution of STs identified in group 1 (toxigenic strain *vacAs1m1*), as defined by PHYLOViZ (goeBURST algorithm) for strains of *H. pylori* isolated from the *M. unguiculatus* animal model.** STs: **ST181** corresponds to the reference strain 26695 of *H. pylori* (*vacAs1m1* toxigenic strain). Each line represents a different allele with mutational changes. The numbers of non-synonymous and synonymous mutations are indicated in parentheses. **ST2902** and **ST2903** were identified at 6 and 12 months and exhibited 5 changes in alleles compared with the initial strain **ST181**.

*Helicobacter pylori* was identified in all infected groups by endpoint PCR. However, *H. pylori* strains were isolated from groups 1 and 3. The genotypes derived from the toxigenic strain (*vacAs1m1/cagA+/cagE+/ babA2+*; **ST181**) in group 1 exhibited genetic alterations at 6 and 12 months (1309 and 2617 G, respectively), and we also observed the emergence of new clones [*vacAs1m1/cagA+/cagE-/babA2-*; **ST2902** (1309 G) and *vacAs1m1/cagA-/cagE+/babA2-*; **ST2903** (2617 G)] (**Figure 1**). Among the seven housekeeping genes that were analyzed, five genes in **ST2902** and **ST2093**, *atpA. efp. mutY. ppa* and *trpC*, had more synonymous mutations. However, the proportions of each gene at 6 and 12 months were similar. The *trpC* gene exhibited the most variation (**Figure 2**).

**FIGURE 3:**
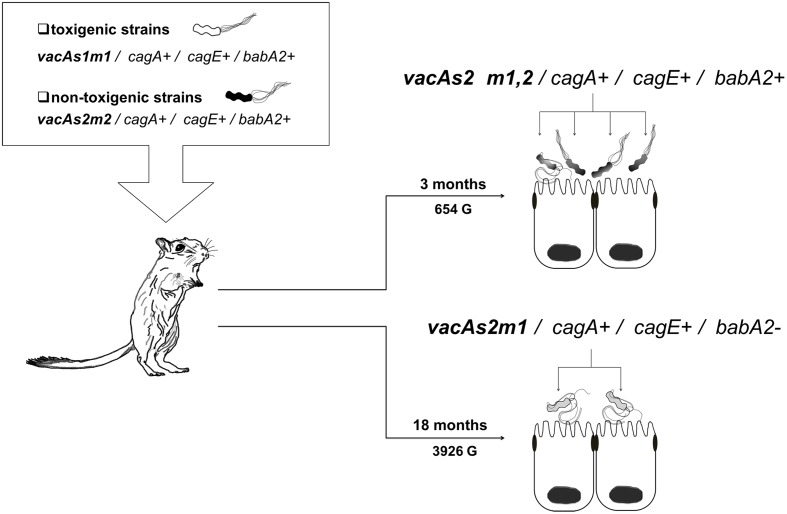
***Helicobacter pylori* genotypes identified in the group 3 toxigenic (*vacAs1m1*) and non-toxigenic (*vacAs2m2*) strains in the *Meriones unguiculatus* animal model.** At 3 months (654 G), both alleles in the middle region of *vacA m1* and *m2* were present (^∗^chimera). At 3 (654 G) and 18 (3926 G) months, alterations in the *babA2* gene were noted.

**FIGURE 4:**
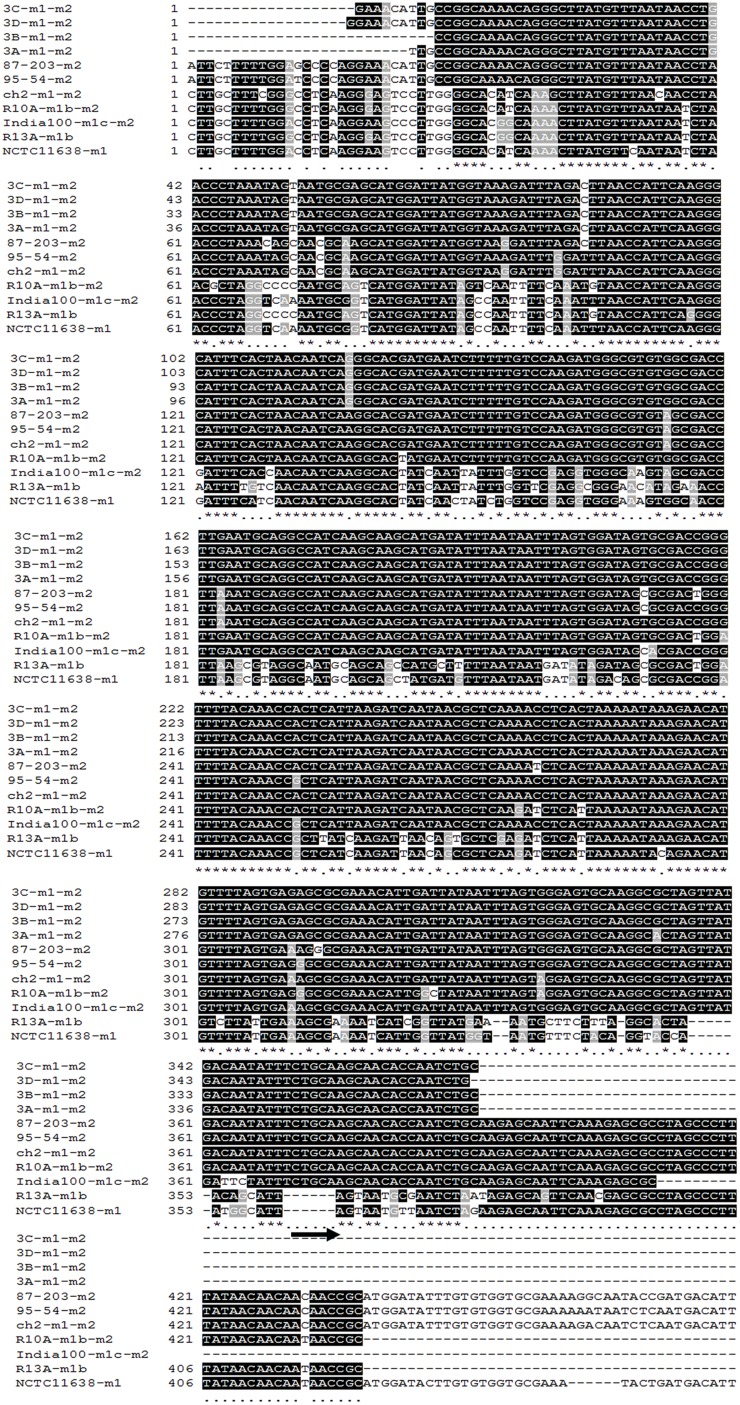
**Alignment of the nucleotide sequences of the middle region of the *vacA* gene of *H. pylori*.** The middle region corresponding to nucleotides (nt) 2308–4400 of the *m1* allele of the reference strain, NCTC11638 (Cover et al., 1994), is presented. Asterisks indicate nucleotides that are identical between the analyzed alleles. Comparisons of *m1-m2* chimeric alleles of *H. pylori* strains obtained from the animal model (strains 3A, 3B, 3C, and 3D), *m2* allele of strain 87–203 (Cover et al., 1994), *m2* allele of strain 95–54 (Pagliaccia et al., 1998), *m1–m2* chimeric alleles of strain ch2 (Ji et al., 2000), *m1-m2* chimeric alleles of strain R10A (Pan et al., 1998), *m1-m2* chimeric alleles of strain India100 (Mukhopadhyay et al., 2000), *m1b* allele of strain R13A (Pan et al., 1998), and *m1* allele of the reference strain, NCTC11638 (Cover et al., 1994). Black stripes identify the portion of the *m1, m2*, and *m1-m2* chimeric alleles (14 nt) in which recombination would have generated chimeric alleles *m1-m2*, as proposed by Pan et al. (1998). The region marked by an arrow identifies the segment that is absent in the *m1* allele but is present in the *m2* allele. The GenBank accession numbers for the presented sequences are **U07145** (NCTC11638), **U05677** (87–203), **U95971** (95–54), **AF191639** (ch2), **AF035609** (R10A), **AF220120** (India100), and **AF035610** (R13A).

In group 3, which was inoculated with the toxigenic (*vacAs1m1/cagA+/cagE+/babA2+*; **ST181**) and non-toxigenic (*vacAs2m2/cagA+/cagE+/ babA2+*; **ST2901**) strains, we also observed genetic alterations at 3 and 18 months (654 G and 3926 G). The strains in this group gave rise to three new clones [*vacAs2m1-2/cagA+/cagE+/babA2+*; **ST2901** (654 G), *vacAs2m1-2/cagA+/cagE+/babA2+*; **ST2904** and *vacAs2m1/cagA+/cagE+/babA2*; **ST2095** (3926 G)] (**Figure 3**). Notably, the genotype identified at 3 months exhibited an alteration in the middle region of the *vacA* gene (*m1-m2*). The nucleotide sequences of the chimera showed 99% and 97% identity with the allelic sequences of *vacAs1m1* and *vacAs2m2*, respectively, from GenBank^6^. In addition, alignment of these chimera sequences with those of other chimeras reported in other studies yielded similar identity values (**Figure 4**).

**FIGURE 5:**
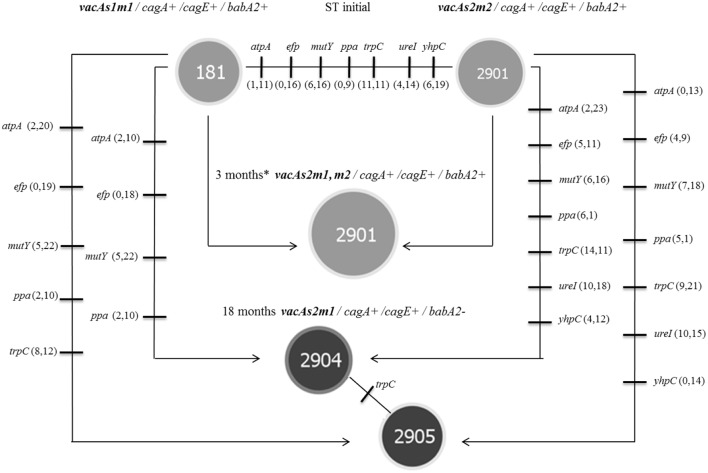
**Microevolution of STs was identified in group 3 (toxigenic strain *vacAs1m1* and non-toxigenic strain *vacAs2m2*), as defined by PHYLOViZ (goeBURST algorithm) for strains of *H. pylori* isolated from the *M. unguiculatus* animal model.** **ST181** and **ST2901** correspond to the reference 26695 (*vacAs1m1* toxigenic strain) and clinical 172F2 (*vacAs2m2* non-toxigenic strain) strains of *H. pylori*, respectively. Each line represents a different allele with mutational changes. The number of non-synonymous and synonymous mutations are indicated in parentheses. **ST2901** was identified at 3 months^∗^ (chimera, **Figure 3**); the data are presented for both original strains. The reference strain 26695 **(ST181)** donated the *vacAm1* allele, which originated the chimera and clinical strain 174F2 **(ST2901)** alleles of the housekeeping genes. The *trpC* gene **(ST2904** and **ST2905)** was the only housekeeping gene with mutational change.

The housekeeping genes of the STs in group 3 exhibited many mutational changes in both the toxigenic and the non-toxigenic strains (**Figure 5**). The number of mutated genes and the number of mutations within each gene were different in the STs. However, the genotype identified at 3 months did not exhibit mutational changes in the housekeeping genes, i.e., the ST was similar to that of the non-toxigenic strain, and the number of synonymous and non-synonymous mutations was similar to that of the toxigenic strain (**ST181**). All non-synonymous mutations occurred outside of the active site and other functionally important sites of the proteins (e.g., signature motif and substrate-binding domains); some synonymous mutations (15.29%) were identified in these regions.

**FIGURE 6:**
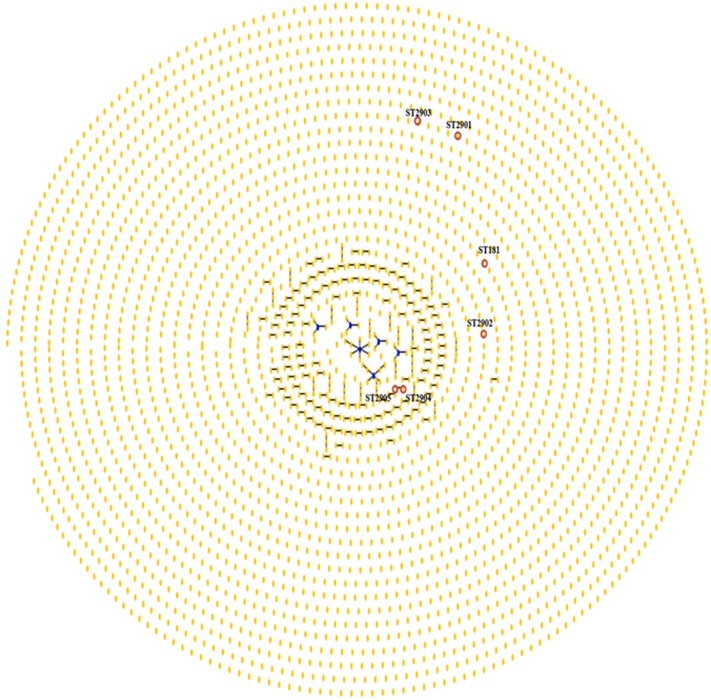
**Population “snapshot” of *H. pylori* and STs found in strains of *H. pylori* that were isolated from the *M. unguiculatus* animal model.** Clusters of related isolates and individual unlinked STs found in the MLST database for *H. pylori* are presented as a single-tree eBURST to define category zero of seven shared alleles. Unions link isolates that correspond to clonal complexes. Primary founders (blue) are located in the center of the group, and founders of subgroups are shown in yellow, as shown for **ST2904** and **ST2905** (red circle). **ST181**, **ST2901**, **ST2902,** and **ST2903** are marked; the labels for the other STs (http://pubmlst.org//helicobacter/) have been removed for clarity.

The phylogenetic analysis of 1993 isolates deposited in the PubMLST database for *H. pylori* along with those isolates of *H. pylori* obtained from the animal model in the present study exhibited an overview of clonal complexes (**Figure 6**). Clusters of related isolates and individual unlinked STs are shown as a single-tree eBURST, establishing the definition of category zero for seven shared alleles. **ST2904** and **ST2905** displayed a ‘double-link’ that featured six alleles in common. Moreover, **ST2901**, **ST2902,** and **ST2903** are individual unlinked STs.

## Discussion

The success of any infection in a host depends on a delicate balance between the host and the pathogen. For bacterial pathogens, the host appears to impose a selective pressure that drives variation within the bacterium (Thompson et al., 2004). In the present study, we infected *M. unguiculatus* with the following *H. pylori* strains with known virulence genotypes and STs: reference strain 26695 (*vacAs1m1*/*cagA+cagE*+*bab2A*+; **ST181**) and clinical strain 172F2 (*vacAs2m2*/*cagA*+*cagE*+*bab2A*+; **ST2901**). Our findings revealed genetic alterations of the introduced genotypes throughout the course of infection in an animal model. Group 1 exhibited genetic alterations in the *cagA, cagE*, and *babA2* genes (**Figure 1**). Several previous studies have observed similar changes or alterations. Solnick et al. (2004) studied genetic alterations in *H. pylori* J166 and found replacements of *babA* with *babB*, suggesting that *babA* undergoes selective pressure early during the course of infection and that changes in *babA* might represent a crucial adaptation to the host stomach environment. In addition, Linz et al. (2014) reported that alterations within the *babA* promoter could occur as early as 1 week post-infection, also they reported mutations in the *cag*-PAI genes in J166 strain after 2 and 6 months in a macaque model, where the function of *cag*-PAI was apparently abolished, implying adaptation by mutation and recombination during early stages of the infection.

More than a decade ago, several authors described the potential for genetic rearrangements and recombination events among *H. pylori* strains during chronic infections in different parts of the stomach (Blaser and Berg, 2001; Blaser and Atherton, 2004). We identified different genotypes at 6 and 12 months post-infection in the group 1 (1309 and 2617 G, respectively) that exhibited alterations in the *cagA, cagE*, and *babA2* genes. In these genes, the incorporation of genetic material by recombination between strains (e.g., *cagA- cagE-* and *babA2-*) was evident. The presence of these genotypes in group 1 after 6 and 12 months strongly suggested that mechanisms of recombination or mutation had occurred over long periods of colonization in our animal model, resulting in the acquisition of this genetic material (**Figure 1**). The same authors suggested that such genetic variation can lead to the development of different strains, genotypes or subclones within the same host. Our data documented mutational changes in housekeeping genes and virulence genotypes; thereby, we identified the following new STs: **ST2902,** **ST2903,** **ST2904,** and **ST2905** derived from **ST181** (group 1) and **ST181** and **ST2901** (group 3). In group 2, which was infected with strain **ST2901** alone, the infection was identified at different periods of time by PCR. However, isolation any *H. pylori* colonies was not possible, and we still do not know why this was the case. Generally, genotype *vacAs2m2* is reported less frequently in infected patients, probably because of its need for more nutrients for its own development *in vitro* or might suggest to be a genotype unsuitable to infect, it can enter to a viable but non-culturable stage (coccoid forms). The coccoid forms of *H. pylori* are less virulent, less likely to colonize and induce inflammation (Mazaheri Assadi et al., 2015). Meanwhile, Pagliaccia et al. (1998) observed *m1* and *m2* alleles of the VacA cytotoxin, which can recognize different receptors on gastric epithelial cells in humans; however, similar findings have not been previously published for gerbils.

Global studies have shown that high degrees of polymorphism in housekeeping genes are associated with changes in the third nucleotide position of codons, and many of the observed variation in STs are synonymous changes (Achtman et al., 1999). We also observed a greater number of synonymous mutations, in accord with other studies (Achtman et al., 1999; Morelli et al., 2010; Secka et al., 2011). In our study, the *trpC* gene (**ST2903**) was the most variable and uniquely introduced stop codon. However, these changes were unlikely to be deleterious for the micro-organisms because they were isolated of animal model. Martincorena et al. (2012) have suggested an evolutionary optimization of the mutation rate to reduce the risk of deleterious mutations.

Phylogenetic studies have indicated a clear separation between sequences of the middle region; the *m2* sequence contains an insertion of 21–25 amino acids that was not present in the *m1* sequence (van Doorn et al., 1998; Gangwer et al., 2007). In the present study, we observed that group 3 (toxigenic strain *vacAs1m1*; **ST181** and non-toxigenic strain *vacAs2m2*; **ST2901**) (**Figure 3**) had a *vacAs2m1-m2/cagA+cagE+babA2+* genotype with the presence of a natural chimera of the middle region of *vacA* at 3 months (654 G). To confirm the presence of the *m1-m2* alleles of *vacA* in the chimera, each allele present in the four strains was sequenced. The identity percentages were 99% (*m1*) and 97% (*m2*), which were similar to those described by Tanih et al. (2011) who reported identity percentages of 87–99 and 89–98% for *m1* and *m2*, respectively, in their chimeras. Our chimeras were aligned with other chimeras that have been previously reported (**Figure 4**), revealing the region proposed by Pan et al. (1998), in which recombination occurs between the *m1* and *m2* alleles in a region with limited homology (14 nt). Natural chimeras in strains of *H. pylori* have rarely been reported, suggesting that *H. pylori* strains with intact *m1* or *m2* of *vacA* provide favorable functional properties and, therefore, exhibit a selective advantage compared with strains containing *m1-m2* chimera sequences (Ji et al., 2000). In the present work, we observed instability of the *vacA m1-m2* chimera; moreover, the allelic combination, *vacAs2m1*, exhibited the best adaptation in the animal model over time. Our results demonstrate the combination of genotypes among the *vacAs1m1* and *vacAs2m2* strains in an animal model. Studies have shown that allelic combination of *vacAs2m1* causes less damage to the host (Atherton et al., 1995). Once *H. pylori* is established in the stomach, it may or may not evolve to a *vacAs1m*1 genotype; however, this genotype is the most frequently noted in adult patients and is associated with duodenal and gastric ulcers and gastric cancer (Atherton et al., 1995, 1997; Miehlke et al., 2000).

The high rate of mutations between STs results in high genetic diversity that reflects a long evolutionary history of various strains of *H. pylori*. In this study, **ST2904** and **ST2905** exhibited seven different housekeeping genes derived from the initial **ST2901**. Thus, we identified four new STs that were reported in the database PubMLST of *H. pylori*. Among these STs, three had alleles that had been previously reported in the database: **ST2901** with the ***mutY*1504** allele reported in Ireland, and **ST2904** and **ST2905** each with an ***atpA*1708** allele reported in Brazil. The phylogenetic analysis conducted with 1993 isolates (PubMLST *H. pylori*; **Figure 6**) provided results that were consistent with previous studies (Suerbaum et al., 1998; Feil and Spratt, 2001; Hanage et al., 2006; Turner et al., 2007), indicating that *H. pylori* forms a non-clonal population, presents a high mutation rate that generates a large number of alleles and that there is a high rate of recombination among these alleles.

The microevolutionary history of *H. pylori* infection in humans reveals remarkable genetic diversity within this bacterium, which is mainly generated by point mutations and recombination (intragenic or intergenic). The high variability of *H. pylori* is thought to maximize its ability to adapt to the changing environment of the host gastric habitat, consequently facilitating chronic colonization. This study provides evidence for processes of recombination between genotypes, the emergence of new clones and patterns of evolutionary non-clonal descent among *H. pylori* strains obtained from an animal model. Our findings suggest that the recombination process in *H. pylori* in the host results from the adaptation of the bacterium to the host.

## Author Contributions

SM-El, GZ, and NV-G conceived and planned the study. SM-El and NA-R performed experiments and generated the database. RL maintained and provided care for the animal model. SM-El, GZ, and NV-G analyzed and interpreted data. PV-M helped with the animal model. SM-Es and FA-H reviewed and corrected the manuscript. The manuscript was prepared by SM-El, GZ, and NV-G. All authors revised and agreed on the final version of the manuscript.

## Conflict of Interest Statement

The authors declare that the research was conducted in the absence of any commercial or financial relationships that could be construed as a potential conflict of interest.

## Footnotes

1 http://pubmlst.org/

2 http://www.expasy.org

3 http://www.softpedia.com/get/Science-CAD/DnaSP.shtml

4 http://www.phyloviz.net/

5 http://eburst.mlst.net/

6 http://www.ncbi.nlm.nih.gov/genbank
